# Psychometric properties of kidney disease quality of life-36 (KDQOL-36) in dialysis patients in Indonesia

**DOI:** 10.1007/s11136-022-03236-6

**Published:** 2022-08-29

**Authors:** M. Rifqi Rokhman, Yulia Wardhani, Dwi Lestari Partiningrum, Barkah Djaka Purwanto, Ika Ratna Hidayati, Arofa Idha, Jarir At Thobari, Maarten J. Postma, Cornelis Boersma, Jurjen van der Schans

**Affiliations:** 1 Department of Health Sciences, Unit of Global Health, University of Groningen, University Medical Center Groningen (UMCG), Ant. Deusinglaan 1, 9713 AV Groningen, The Netherlands; 2 Institute of Science in Healthy Ageing & healthcaRE (SHARE), University Medical Center Groningen, University of Groningen, Groningen, The Netherlands; 3 Faculty of Pharmacy, Universitas Gadjah Mada, Yogyakarta, Indonesia; 4 Faculty of Medicine, Public Health and Nursing, Universitas Gadjah Mada, Yogyakarta, Indonesia; 5 Faculty of Medicine, Universitas Diponegoro, Semarang, Indonesia; 6 Faculty of Medicine, Universitas Ahmad Dahlan, Yogyakarta, Indonesia; 7 Department of Pharmacy, Faculty of Health Science, Universitas Muhammadiyah Malang, Malang, Indonesia; 8 Dr. Syaiful Anwar Hospital, Malang, Indonesia; 9 Department of Economics, Econometrics and Finance, Faculty of Economics & Business, University of Groningen, Groningen, The Netherlands; 10 Unit of PharmacoTherapy, Epidemiology and Economics (PTE2), Department of Pharmacy, University of Groningen, Groningen, The Netherlands; 11 Department of Pharmacology and Therapy, Faculty of Medicine, Universitas Airlangga, Surabaya, Indonesia; 12 Center of Excellence in Higher Education for Pharmaceutical Care Innovation, Universitas Padjadjaran, Bandung, Indonesia; 13 Faculty of Management Sciences, Open University, Heerlen, The Netherlands

**Keywords:** Validation, Quality of life, KDQOL, SF-12, Indonesia

## Abstract

**Objective:**

The study aimed to evaluate the psychometric properties of KDQOL-36 Bahasa Indonesia in hemodialysis (HD) and continuous ambulatory peritoneal dialysis (CAPD) patients in Indonesia.

**Methods:**

The psychometric analysis was conducted in three hospitals offering both HD and CAPD. The validity was assessed through structural, convergent, and known-group validity, while reliability was evaluated using internal consistency and test–retest reliability.

**Results:**

The study involved 370 participants of which 71% received HD treatment. No floor and ceiling effects (< 10%) were identified. Confirmatory factor analysis supported a good model fit for both generic and kidney-specific domains, while exploratory factor analysis revealed three factors for kidney-specific domains and only three items with a loading factor below 0.4. Convergent validity showed positive correlations between kidney-specific domains, generic domains, and EQ-5D. The comparison of quality of life among subgroups based on dialysis type and whether or not patients had diabetes supported the hypotheses of known-group validity. Cronbach’s alpha and omega values had demonstrated good internal consistency. Test–retest reliability indicated burden of kidney disease had good reliability, while other domains had moderate reliability.

**Conclusion:**

The study supports the validity and reliability of both generic and kidney-specific domains of KDQOL-36 Bahasa Indonesia to evaluate quality of life in patients with HD and CAPD in Indonesia. As health-related quality of life is a crucial predictor of patient outcomes, this report contributes new evidence about validity and reliability to recommend the use of KDQOL-36 Bahasa Indonesia in dialysis centers.

**Supplementary Information:**

The online version contains supplementary material available at 10.1007/s11136-022-03236-6.

## Introduction

Extending patients’ life is the ultimate goal of patient care support, but enhancing the quality of patients’ life is also of interest [1]. Patients with end-stage renal disease need renal replacement therapy either in the form of dialysis or renal transplantation [2]. Renal transplantation remains the best treatment option for end-stage renal disease patients resulting in better quality of life and overall survival [3, 4]. However, there are practical challenges in increasing uptakes of renal transplants due to a scarcity of donated kidneys. Consequently, treatment with dialysis seems to be a feasible first-line treatment option [5].

Two dialysis modalities are offered in Indonesia in the forms of hemodialysis (HD) and continuous ambulatory peritoneal dialysis (CAPD). A previous modeling study demonstrated that CAPD is more cost-effective compared to HD for the Indonesian setting [6]. In addition, a recent meta-analysis also showed that although there were no significant differences in quality of life between HD and peritoneal dialysis treatment, more patients with peritoneal dialysis had a better quality of life [7]. Nevertheless, the use of HD (98%) predominated over CAPD (2%) in 2018 in Indonesia, and the increase of patients using CAPD treatment was not significant from year to year [8]. A questionnaire with acceptable psychometric properties is required to assess and compare quality of life in patients undergoing HD and CAPD treatment.

Several large studies, such as a study in North America and an international prospective study conducted in five European countries, Japan and the USA, have demonstrated that poor health-related quality of life (HRQOL) could independently predict death and hospitalization of dialysis patients [9, 10]. Consequently, HRQOL has been suggested to be used as a valuable supplement to clinical outcome measures [9]. Therefore, an instrument to measure HRQOL of patients with dialysis is crucial. The kidney disease quality of life (KDQOL) is a kidney disease-specific HRQOL instrument, and the KDQOL-36 version is a preferred measurement tool and widely used in dialysis facilities because of its ease of administration with a relatively minimal burden both on patients and staff [11]. The KDQOL-36 questionnaire is developed in English and has been translated and cross-culturally validated in many countries [12]. A recent systematic review on psychometric properties of KDQOL-36 found that the instrument is recommended for the assessment of quality of life in patients with dialysis [13]. Nonetheless, the review found inconsistency and low quality of evidence for psychometric properties of KDQOL-36, such as in structural validity and internal consistency, and further studies are needed to examine its psychometric properties [13].

Three previous studies had translated (using forward–backward translation) and cross-culturally validated the Indonesian version of KDQOL-36 [14–16]. However, these studies reported the psychometric properties only in patients with HD. Since there is an item in the KDQOL-36 that is specifically intended for peritoneal dialysis patients, the Indonesian version of KDQOL-36 should also be tested in CAPD patients. Furthermore, all these three studies were conducted only in a single hospital, and the number of samples was not enough to analyze the structural validity using confirmatory factor analyses. Patients with HD in various settings of HD centers may have different severity levels and demographic heterogeneity. Thus, additional evidence of the psychometric properties of the Indonesian version of KDQOL-36 in a broader patient population undergoing dialysis and different settings needs to be gathered.

Therefore, this study aimed to evaluate validity and reliability of KDQOL-36 Bahasa Indonesia both in HD and CAPD patients.

## Methods

### Study setting

The study was conducted in Yogyakarta (Dr. Sardjito General Hospital and PKU Muhammadiyah Bantul Hospital) and Malang (Dr. Syaiful Anwar Hospital), Indonesia from January to June 2021. These three hospitals were selected since they offered both HD and CAPD treatments.

### Participants

All patients receiving HD and CAPD were recruited to participate in the study. The inclusion criteria of the participants were patients aged ≥ 18 years old, diagnosed with end-stage renal disease and undergoing HD or CAPD treatment for at least three months, able to speak Indonesian, and agreeing to participate in the study. The exclusion criteria were patients with mental illness or cognitive impairment.

The minimum sample size was determined based on a number of guidelines. A sample of 100 subjects in each HD and CAPD group was able to detect a statistically significant difference between groups by independent *t*-tests based on 80% power (*p* = 0.05, two-tailed) with Cohen’s effect size of 0.4 [17]. In order to confirm the structural validity using confirmatory factor analysis, a minimum sample size of 315 participants (with missing data) or 265 participants (without missing data) was needed for a power of 0.80 [18]. Another consideration, based on the ratio of the number of items and participants to perform factor analysis, the minimum number of participants required to validate KDQOL-36 with 36 items was 360 participants (the number of items multiplied by 10) [19]. Therefore, the minimum number of participants in the study was 360 participants and each HD and CAPD group had to have at least 100 participants.

### Study instruments

The study instrument contained the participants’ characteristic form, the Indonesian version of KDQOL-36 and the EuroQoL 5-Dimension 5-Level (EQ-5D-5L). Our study used the Indonesian version of KDQOL-36 translated by Cahyono et al. (2018) since the translation was already available using forward–backward translation from two independent translators in each step according to the standard translation process [15]. However, since Cahyono et al. (2018) did not translate item no 28b (specific item for patients with peritoneal dialysis), we obtained this item from Hidayah (2016).

KDQOL-36 contains both generic and kidney-specific domains. The first 12 items are generic domains assessed using the 12-Item Short-Form Health Survey (SF-12) instrument that can be scored to obtain the physical component score (PCS) and the mental component score (MCS). The last 24 items are kidney-specific domains that can be used to quantify burden (4 items), symptoms (12 items), and effects (8 items) of kidney disease [12]. Item number 28a of the KDQOL-36 questionnaire should only be answered by HD patients, while item number 28b is only intended for peritoneal dialysis patients. The scores of the KDQOL-36 questionnaire are transformed into a score between 0 to 100, with higher scores reflecting better quality of life [20].

### Study procedure

The standard translation process consists of translation, pilot-testing, and psychometric analysis to estimate the validity and reliability [21, 22]. In order to conform to this standard, before the psychometric analysis of KDQOL-36 Bahasa Indonesia, recommendations suggested the involvement of two to six experts in the pilot-testing process [23]. In this study, five experts (consisting of two nephrologists, an academician experienced in the validation of instruments, and two dialysis nurses) assessed the clarity of each item of KDQOL-36 Bahasa Indonesia. Clarity means that items can be clearly described without confusion [24, 25]. The clarity scale was “clear” and “not clear”. If an expert stated that an item was not clear, additional recommendations by the expert were required.

After this step, interviews were conducted with ten patients undergoing dialysis with different education levels, balanced for the number of HD/CAPD patients and age to assess the clarity and interpretation of each item. The participants were asked whether they could understand each item and explain the meaning of each item using their own words [26]. Based on pilot testing from experts and patients, three items were revised, namely item number 18 (from “Sakit dada?” to “Nyeri dada?”), item number 28b (from “Masalah dengan jalur/tempat masuknya kateter Anda?” to “Masalah di sekitar perut Anda tempat masuknya kateter?”), and item number 35 (from “Kehidupan hubungan intim Anda?” to “Aktivitas seks Anda?”).

Measurement of psychometric properties was conducted by distributing the instrument to at least 360 participants to assess the validity and reliability (internal consistency) of KDQOL-36 Bahasa Indonesia in three hospitals. Test–retest reliability was also conducted to assess reliability by repeating the measurement process on the same subjects after 2 weeks in at least 30 patients [17, 27].

### Statistical analysis

Descriptive statistics were used to compare the socio-demographic characteristics of patients on HD and CAPD. Differences in characteristics between groups were tested using the *χ*^2^ test for categorical variables, independent *t*-tests were used for continuous variables with normal distribution, or Mann–Whitney tests for not-normally distributed continuous variables.

The KDQOL™-36 scoring program (v.20) was used for scoring PCS, MCS, and kidney-specific domains (burden, symptoms, and effects of kidney disease). The KDQOL™-36 scoring program (v.20) is designed as an Excel spreadsheet, consisting of five sheets: Raw, Convert, Score, Scale, and Stats, developed by RAND Health Care, while the copyright was owned by UCLA Division of General Internal Medicine and Health Services Research [12].

The validity was assessed by structural, convergent, and known-group validity. A confirmatory factor analysis was used to confirm the structural validity, and model fit was determined based on the model’s Chi-squared statistic (*χ*^2^), the root mean square error of approximation (RMSEA), the comparative fit index (CFI) and Tucker-Lewis index (TLI). A non-significant Chi-squared statistic, lower value of RMSEA, higher CFI and TLI indicate better goodness-of-fit. Confirmatory factor analysis indicated acceptable fit if the Chi-squared statistic was non-significant, RMSEA < 0.07 (sample size more than 250 participants), CFI and TLI > 0.95 [28]. Nonetheless, when sample size is large enough, the Chi-squared statistic is likely to be significant and leads to the rejection of models even when the residuals are very small and the model has good model fit.

The KDQOL-36 items have ordered categorical responses; therefore, confirmatory factor analysis was evaluated using the diagonally weighted least squares estimator. The analysis was conducted using the lavaan package in R [29]. The generic and kidney-specific disease domains were analyzed separately in confirmatory factor analysis. Based on the previous publications, the generic domains of KDQOL-36 have a good fit for two latent variables (PCS and MCS) [30, 31], while kidney-specific disease domains have three latent variables (burden, symptoms, and effects of kidney disease) [17, 32]. Each latent variable was allowed to correlate with one another. Variances for latent variables were set to one, while loading factors on other domains were fixed to zero (Supplements 1 and 2). The results were reported based on standardized parameter estimates.

Exploratory factor analysis of kidney-specific domains was also carried out. A loading factor of > 0.4 indicates a good relationship between an item and the underlying factor [19], while a loading factor in the range of 0.30–0.40 meets the minimal level for interpretation of structure [28]. Exploratory factor analysis was conducted using the psych package in R, and the weighted least squared and polychoric correlations were used to estimate exploratory factor analysis [33]. The number of factors to be extracted was determined using the parallel analysis (Supplement 3).

The convergent validity was assessed using Pearson’s correlation. Since both the kidney-specific domains, generic domains, and EQ-5D measure different aspects of HRQOL, we hypothesized that the correlations would be positive and weak to moderate. The EQ-5D index score was calculated using the Indonesian value set [34]. The correlation was classified as very weak (< 0.20), weak (0.20–0.39), moderate (0.40–0.59), strong (0.60–0.79), and very strong (> 0.80) [35]. Known-group validity was assessed by comparing scores on generic and kidney-specific domains between subgroups based on dialysis type (patients undergoing CAPD were hypothesized to have better HRQOL than HD), and whether the patient had diabetes (patients with diabetes were hypothesized to have lower HRQOL than patients without diabetes) [36]. The effect sizes were calculated and classified according to Cohen as small (0.2), medium (0.5), or large (0.8) [37].

Reliability was assessed using the test–retest reliability and internal consistency [17]. Test–retest reliability was assessed using intraclass correlation coefficients (ICC), and ICC should be reported including the following items: model, type, and definition selections [38]. In this study, ICC was measured based on the test–retest method, so ICC was calculated using a two-way mixed-effects model, single rater, and absolute agreement. An ICC value between 0.5 and 0.75 is considered as moderate and 0.75–0.9 as good [38]. The difference between the baseline and two-week retest was assessed using paired *t*-tests. A domain with a Cronbach’s alpha value ≥ 0.7 indicates acceptable internal consistency [19]. The Cronbach’s alpha values were not calculated for PCS and MCS due to the nature of scoring for SF-12 and items with different level options [32].

Besides a Cronbach’s alpha, McDonald’s omega hierarchical (*ω*_*h*_) and total (*ω*_*t*_) were reported to estimate internal consistency. Omega was estimated using the psych package in R [39]. Although there is no generally accepted guideline to determine the minimum levels of omega for clinical decision-making [40], *ω*_*t*_ value should meet the same criteria as Cronbach’s alpha standard (≥ 0.7). Similarly, *ω*_*h*_ value should be at least 0.50 but 0.8 would be preferred [40, 41]. The main benefit of using omega over Cronbach’s alpha is that omega is estimated within a factorial model and represents more realistic assumptions [42].

Percentages of ceiling and floor effects were assessed. Ceiling effects are estimated as being the percentage of respondents with scores of 100, while floor effects are the percentage of respondents having a score of 0. Ceiling and floor effects should be less than 20% to ensure that the scale captures the full range of potential responses within the population, and that changes over time can be detected [43].

All statistical analysis was performed in SPSS Version 26.0, except for factor analysis and omega estimation, which used R. A *p*-value lower than 0.05 was considered a significant difference.

### Ethics statement

Ethical approval was obtained from the Medical and Health Research Ethics Committee (MHREC), Faculty of Medicine, Public Health and Nursing of Universitas Gadjah Mada–Dr. Sardjito General Hospital with document number KE/FK/0953/EC/2020 on 27 August 2020. After explaining the aims and procedures of the study, written informed consent was obtained from all prospective participants who agreed to participate. A copy of the participatory information and informed consent sheet was given to all participants.

## Results

The questionnaires were distributed to 383 prospective participants, but 13 participants refused to participate in the study (response rate = 96.6%). In the end, a total of 370 participants participated in the study, of which 262 patients (71%) received HD treatment and 108 patients (29%) received CAPD (Table 1). There was a significant difference in the socio-demographic characteristics between patients with HD and CAPD, except for gender (*p* = 0.715) and duration of dialysis (*p* = 0.300). Patients with CAPD were younger (43.1 vs. 51.9, *p* < 0.001), higher in educational level (41% vs. 24% having a diploma degree or higher, *p* = 0.001), higher proportion of non-married status (21% vs. 10%, *p* = 0.002), higher proportion of participants who were still working (48% vs. 29%, *p* = 0.001), and lower proportion with diabetes (15% vs. 29%, *p* = 0.003) than HD patients.

**Table 1:** Descriptive characteristics of study participants (n = 370)

Socio-demographic	Total	HD patients (*n* = 262)	CAPD patients (*n* = 108)	*p*-value^a^
Age, mean (SD), year (*n* = 367)	49.4 (13.4)	51.9 (12.2)	43.1 (14.5)	< 0.001
Gender, *n* (%)				
Male	238 (64.3)	167 (63.7)	71 (65.7)	0.715
Female	132 (35.7)	95 (36.3)	37 (34.3)	
Educational background, *n* (%)				
Elementary school or lower	79 (21.4)	66 (25.2)	13 (12.0)	0.001
Junior or senior high school	185 (50.0)	134 (51.1)	51 (47.2)	
Diploma or higher	106 (28.6)	62 (23.7)	44 (40.7)	
Marital status, *n* (%)				
Single	48 (13.0)	25 (9.5)	23 (21.3)	0.002
Married	322 (87.0)	237 (90.5)	85 (78.7)	
Working, *n* (%)				
Yes	129 (34.9)	77 (29.4)	52 (48.1)	0.001
No	241 (65.1)	185 (70.6)	56 (51,9)	
Having a diabetes, *n* (%)				
Yes	93 (25.1)	77 (29.4)	16 (14.8)	0.003
No	277 (74.9)	185 (70.6)	92 (85.2)	
Duration of dialysis, mean (SD), year (n = 367)	4.3 (3.6)	4.6 (3.9)	3.8 (2.7)	0.300

*HD* hemodialysis; *CAPD* continuous ambulatory peritoneal dialysis; *SD* standard deviation

^a^*p*-values were obtained from the differences between HD and CAPD patients

### Validity of KDQOL-36 Bahasa Indonesia

**Fig. 1:**
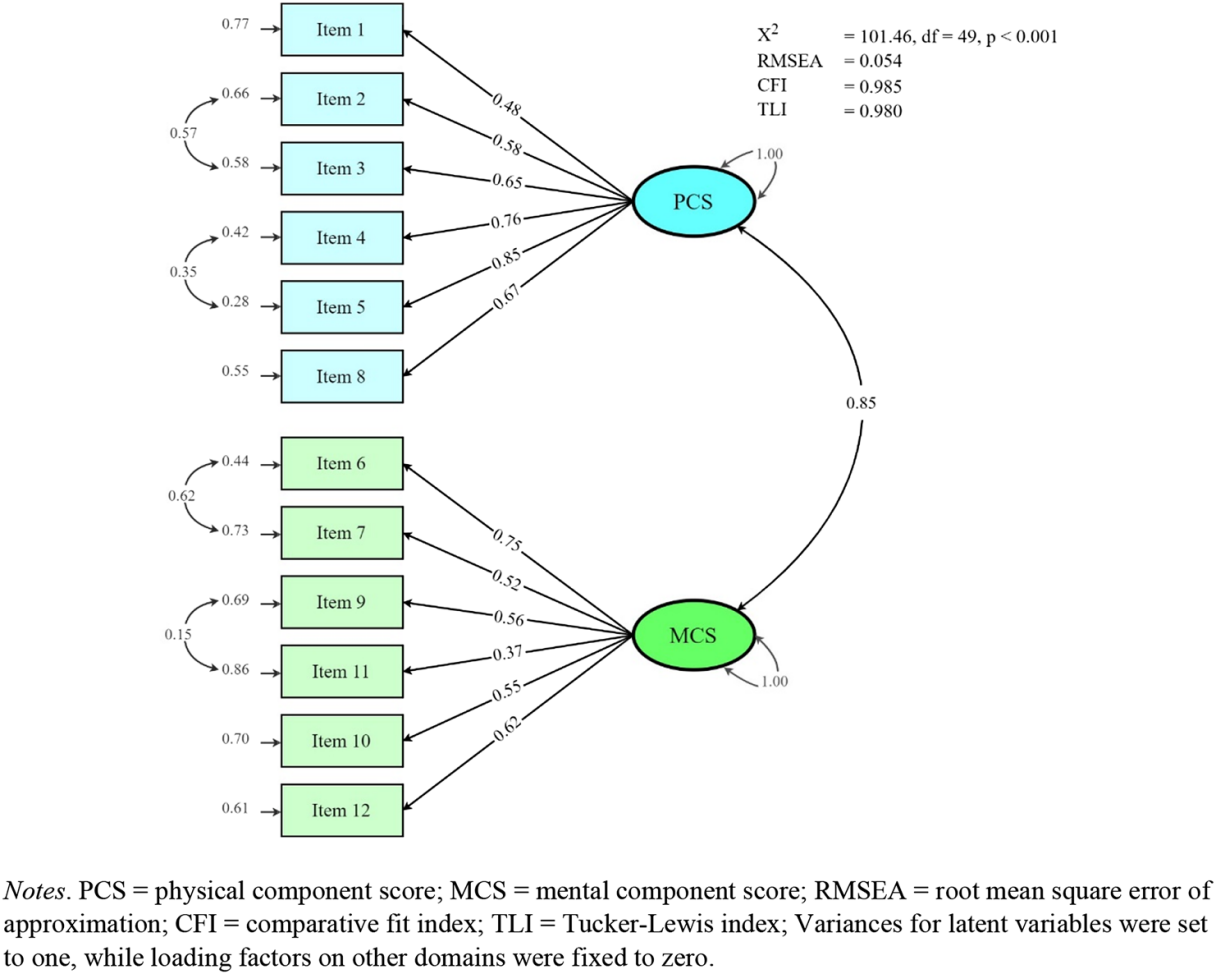
Confirmatory factor analysis of generic domains (SF-12) of KDQOL-36 Bahasa Indonesia

**Fig. 2:**
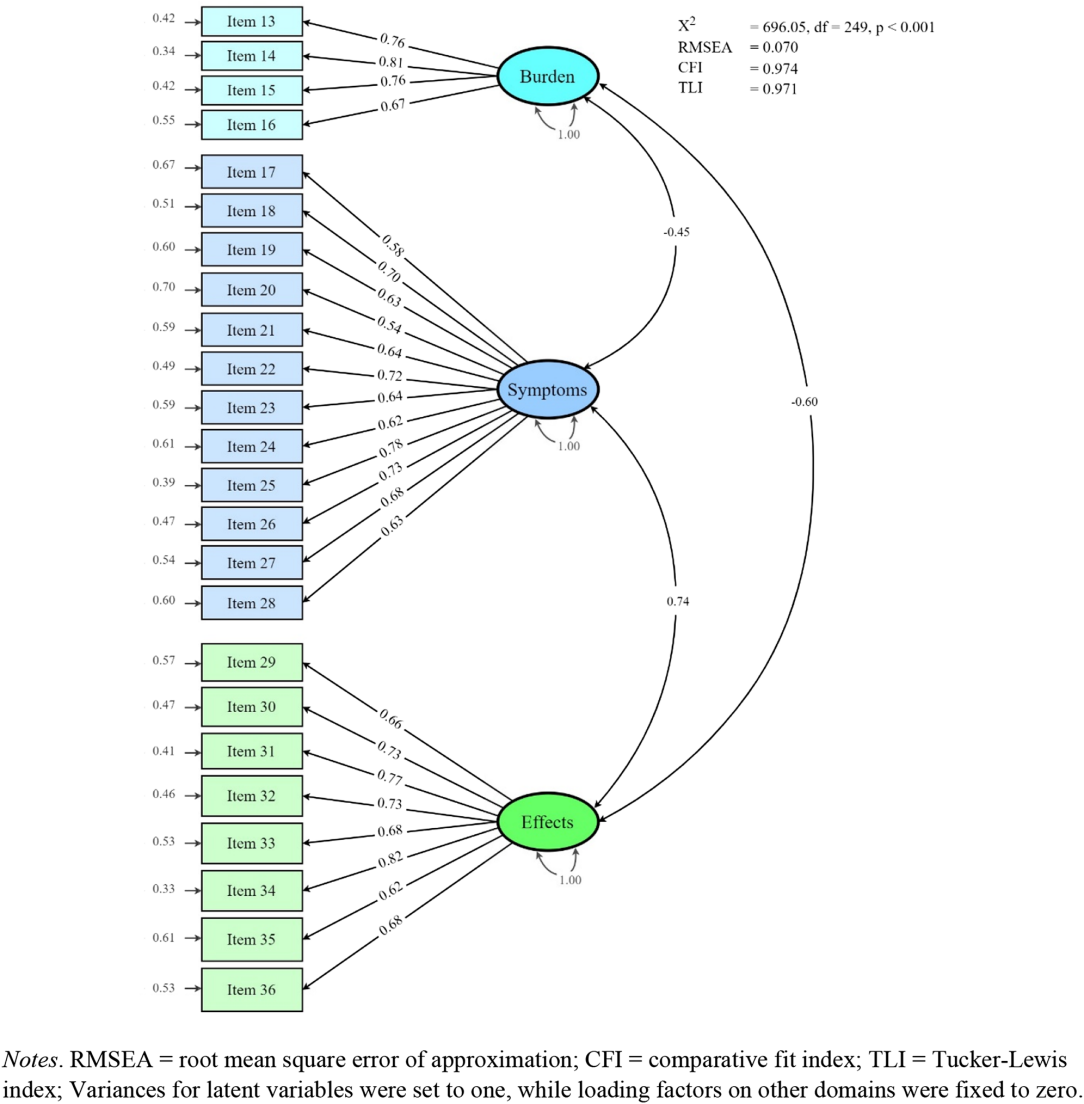
Confirmatory factor analysis of kidney disease domains of KDQOL-36 Bahasa Indonesia

Confirmatory factor analysis showed high goodness-of-fit for generic domains with an *χ*^2^ = 101.46 (*p*-value < 0.001), RMSEA value of 0.054, CFI of 0.985 and TLI 0.980 when specified with covariations between the error of the items that belong to the same subdomains (Fig. 1). The model had lower goodness-of-fit parameters when run without covariations (Supplement 1). For kidney-specific domains, the model had high goodness-of-fit indicated by an *χ*^2^ = 696.05 (*p*-value < 0.001), RMSEA of 0.070, CFI of 0.974 and TLI 0.971 (Fig. 2).

Based on parallel analysis, three factors were suggested to be extracted, and exploratory factor analysis was examined using promax rotation. Four items had a high correlation with the third factor (burden of kidney disease), 12 items with the first factor (symptoms of kidney disease), and 8 items with the second factor (effects of kidney disease). All items had a loading factor of more than 0.4 and were between the range of 0.40–0.99, and only three items (items number 17, 34, and 36) had a loading factor below 0.4 (Table 2). From these three items, the loading factors of two items (number 34 and 36) were still higher than 0.3 but one item (number 17) was slightly lower than 0.3.

**Table 2:** Exploratory factor analysis of kidney-specific domains of KDQOL-36 Bahasa Indonesia

No.	Items	Domain	Factor loadings^a^		
			1	2	3
13	Interfered by my kidney disease	Burden of kidney disease	0.19	0.28	**0.59**
14	Too much time spent on my kidney disease		0.22	0.30	**0.68**
15	Frustrated with my kidney disease		0.16	0.11	**0.71**
16	Burden on my family		0.08	0.22	**0.85**
17	Soreness in muscles	Symptoms or problem lists	**0.29**	0.25	0.10
18	Chest pain		**0.80**	0.14	0.05
19	Cramps		**0.78**	0.18	0.02
20	Itchy skin		**0.48**	0.08	0.04
21	Dry skin		**0.50**	0.13	0.03
22	Shortness of breath		**0.75**	0.02	0.07
23	Faintness or dizziness		**0.78**	0.24	0.11
24	Lack of appetite		**0.68**	0.05	0.15
25	Washed out or drained		**0.66**	0.05	0.23
26	Numbness in hands or feet		**0.67**	0.09	0.02
27	Nausea or upset stomach		**0.55**	0.18	0.03
28	Problems with access or catheter site		**0.63**	0.09	0.10
29	Fluid restriction	Effects of kidney disease	0.12	**0.64**	0.10
30	Dietary restriction		0.28	**0.55**	0.11
31	Ability to work around the house		0.25	**0.99**	0.01
32	Ability to travel		0.15	**0.82**	0.05
33	Dependent on doctors and other medical staff		0.32	**0.43**	0.04
34	Stress or worries caused by kidney disease		0.32	**0.35**	0.23
35	Sex life		0.23	**0.40**	0.05
36	Personal appearance		0.28	**0.30**	0.19
		% of variance	23.6	14.1	9.4

Extracted method: weighted least squares estimation; Rotation method: promax

^a^Higher factor loadings indicate a greater correlation between an item and the underlying concept

Pearson's correlations between kidney-specific domains with PCS, MCS, and EQ-5D index score showed positive correlations from weak to moderate (0.32–0.47) (Table 3). A very weak correlation was found between PCS and MCS with Pearson’s correlation value of 0.05.

**Table 3:** Convergent validity between KDQOL-36 Bahasa Indonesia and EQ-5D

Domain	PCS	MCS	Burden	Symptoms	Effects	EQ-5D index score
Generic domains (SF-12)						
PCS	1					
MCS	0.05	1				
Kidney-specific domains						
Burden of kidney disease	0.32**	0.35**	1			
Symptoms of kidney disease	0.34**	0.40**	0.37**	1		
Effects of kidney disease	0.47**	0.41**	0.48**	0.64**	1	
EQ-5D index score	0.48**	0.27**	0.34**	0.40**	0.45**	1

*PCS* physical component score; *MCS* mental component score; *HD* hemodialysis; *CAPD* continuous ambulatory peritoneal dialysis

**Correlation is significant at the 0.01 level; Higher values between measures indicate higher correlations

In all domains, patients with CAPD had better HRQOL than HD except for burden of kidney-specific domains where patients with HD had slightly better HRQOL (Table 4). The differences were only statistically significant in PCS (*p* = 0.043) and effects of kidney disease (*p* = 0.014) with effect sizes 0.11 and 0.13, respectively. Moreover, patients without diabetes also had significantly better HRQOL than patients with diabetes in all domains, except for MCS domain (*p* = 0.184). These findings supported known-group validity. In all domains that have significant differences, the effect sizes were considered small (< 0.2), and only in PCS and effects of kidney disease, the effect sizes were higher than 0.2.

**Table 4:** Known-group validity of KDQOL-36 Bahasa Indonesia

	PCS	MCS	Burden	Symptoms	Effects
Dialysis type					
HD	38 (9.9)	47.7 (10)	51.7 (24.7)	75 (18.9)	71.3 (20.7)
CAPD	40 (9)	49 (9)	49.1 (23.8)	77.9 (13.5)	77.6 (16.1)
*p*-value^a^	0.043	0.309	0.346	0.691	0.014
Effect size	0.11	0.05	0.05	0.02	0.13
Diabetes					
Yes	33.7 (9.4)	47 (10.3)	45.4 (23.3)	72 (17.5)	64.1 (19.4)
No	40.2 (9.3)	48.5 (9.5)	52.8 (24.6)	77.2 (17.4)	76.2 (18.8)
*p*-value^a^	< 0.001	0.184	0.005	0.008	< 0.001
Effect size	0.30	0.07	0.15	0.14	0.28

*PCS* physical component score; *MCS* mental component score; *HD* hemodialysis; *CAPD* continuous ambulatory peritoneal dialysis, *SD* standard deviation

^a^*p*-values were obtained from the differences between subgroups based on dialysis type or whether the patient had diabetes; Higher scores of each domain of KDQOL-36 Bahasa Indonesia in HD and CAPD patients indicate better quality of life

### Reliability of KDQOL-36 Bahasa Indonesia

Reliability was assessed through ICC, omega, and Cronbach’s alpha values (Table 5). Test–retest reliability was carried out in 40 participants (30 HD and 10 CAPD patients) and no significant differences were measured between the baseline and 2-week retest for all domains of the KDQOL-36 Bahasa Indonesia. ICC values indicated that both generic and specific-kidney disease had moderate reliability (ICC values ranged between 0.56 and 0.73) and one domain, burden of kidney disease, had good reliability (ICC value = 0.79). All ICC values were higher than 0.7, except for the PCS (ICC value = 0.56). The ω_h_ and ω_t_ values for generic domains were 0.62 and 0.84, while the values were 0.56 and 0.92 for kidney-specific domains of KDQOL-36 Bahasa Indonesia. All kidney-specific domains of KDQOL-36 Bahasa Indonesia had a Cronbach’s alpha value higher than 0.7. This indicated good internal consistency. No significant floor and ceiling effects (< 10%) were found in all five domains of KDQOL-36 Bahasa Indonesia.

**Table 5:** Reliability of KDQOL-36 Bahasa Indonesia

Domain	Mean (SD)		*p*-value^a^	Effect size	ICC^b^	Floor^c^ *n* (%)	Ceiling^c^ *n* (%)	Cronbach's alpha^d^	
	Baseline	2-week retest						HD	CAPD
Generic domains (SF-12)									
Physical component score	38.9 ± 8.5	38.6 ± 9.5	0.936	0.01	0.56	–	–	–	–
Mental component score	48.2 ± 10.8	49.9 ± 10.7	0.185	0.15	0.73	–	–	–	–
Kidney-specific domains									
Burden of kidney disease	50.3 ± 25.9	51.3 ± 27.5	0.736	0.08	0.79	10 (2.7)	13 (3.5)	0.75	0.79
Symptoms of kidney disease	76.1 ± 17.6	79.7 ± 15.3	0.104	0.18	0.72	–	11 (3.0)	0.88	0.79
Effects of kidney disease	72.3 ± 15.7	73.4 ± 18.1	0.409	0.09	0.72	2 (0.5)	28 (7.6)	0.85	0.81

*PCS* physical component score; *MCS* mental component score; *SD* standard deviation; *ICC* intraclass correlation coefficient

^a^*p*-values were obtained from the differences between baseline and retest in 40 patients

^b^ICC was calculated using a 2-way mixed-effects model, single rater, and absolute agreement

^c^Floor and ceiling effects were computed from 370 patients

^d^Cronbach’s alpha values were computed from 262 HD patients and 108 CAPD patients; Higher ICC and Cronbach’s alpha values indicate higher reliability

## Discussion

KDQOL-36 is the most common instrument used to evaluate HRQOL of patients with dialysis. This is the first study that conducted an analysis of psychometric properties of the KDQOL-36 Bahasa Indonesia in patients treated with both HD and CAPD in Indonesia. Overall, KDQOL-36 Bahasa Indonesia has adequate validity and reliability to measure quality of life in patients undergoing both HD and CAPD treatments.

KDQOL-36 Bahasa Indonesia shows desirable structural validity. The findings from both confirmatory and exploratory factor analysis are consistent with previous studies and support the structural validity of KDQOL-36 [44, 45]. Goodness-of-fit for confirmatory factor analysis of generic domains improved when it was specified with covariations between the error of the items that belong to the same subdomains. A current systematic review that analyzed the psychometric properties of the KDQOL-36 instrument found very low quality of evidence in structural validity since it was performed only in kidney-specific domains, assessing not all items of KDQOL-36, and the sample size was less than five times the number of items [13]. Our study assessed the structural validity of all five domains of KDQOL-36 and had a sufficient sample size to conduct a confirmatory factor analysis, which can be used as additional evidence to strengthen the structural validity of the KDQOL-36 instrument.

In general, our findings support the hypotheses on convergent validity, that kidney-specific domains are correlated with PCS, MCS and EQ-5D scores. In addition, the results of factor analysis also support structural validity of both generic and kidney-specific disease domains. However, the correlation between PCS and MCS based on Pearson’s correlation was different compared to its correlation in confirmatory factor analysis due to the difference in the scoring algorithm to derive PCS and MCS [46]. The standard scoring algorithm for PCS and MCS is derived based on an uncorrelated (orthogonal) factor model [47, 48]; therefore, the correlation between PCS and MCS in our study was very weak based on Pearson’s correlation (0.05) as found in other studies [17, 49, 50]. On the other hand, the correlation between PCS and MCS in our confirmatory factor analysis was allowed. Consequently, the correlation was high (0.85) as supported by other studies [30, 43, 44].

The results of known-group validity are in accordance with prior hypotheses. The comparison of HRQOL based on dialysis type showed that the effects of the kidney disease domain are sensitive and can discriminate between HD and CAPD patients, although the effect sizes were small. This finding is supported by a previous study conducted in China [17]. In Indonesia, HD patients spend 2–3 times a week visiting a hospital for receiving dialysis and 4 h each visit excluding travel time, but CAPD patients only have to visit a hospital once in a month. Therefore, CAPD patients compared to HD patients may feel that their kidney disease has less “dependence on doctors and other medical staff” and patients have more chance and “ability to travel”.

Previous research recommends that omega as model-based reliability estimates is more realistic assumptions than Cronbach’s alpha and properly estimates reliability for multidimensional tests [40, 42]. However, to the best of our knowledge, no existing studies analyzed the psychometric properties of KDQOL-36 reporting omega. A recent systematic review assessing the psychometric properties of KDQOL-36 also did not assess omega as part of internal consistency parameter [13]. Omega total represents the combined reliability of all factors in the model without distinguishing between specific and general sources of variance, whereas omega hierarchical value is used to correctly estimate the general factor's reliability by controlling the variance of the specific factors [51]. In our study, omega total values for both generic and kidney-specific domains of KDQOL-36 Bahasa Indonesia were higher than the standard (0.7), while the omega hierarchical values were higher than 0.5. We suggest that future studies report these omega values so that reliability can be estimated more accurately and can be compared between studies.

In Indonesia, all dialysis treatments in terms of HD and CAPD are reimbursed under the national insurance scheme. KDQOL-36 Bahasa Indonesia as a questionnaire with adequate validity and reliability can be used to examine these differences in quality of life between patients with HD and CAPD. This finding could be used to convince both healthcare professionals and patients to promote the uptake of CAPD in Indonesia. Moreover, this questionnaire can also be used by dialysis centers as a supplement to clinical outcome measures since previous studies have confirmed the correlation between KDQOL-36 scores with mortality and hospitalization [10].

This study included a number of hospital settings in terms of hospital type and class. Data was collected from two class A public hospitals (Dr. Sardjito General Hospital, Dr. Syaiful Anwar Hospital) and one class C private hospital (PKU Muhammadiyah Bantul Hospital). Despite this strength, several limitations need to be discussed. The test–retest study to analyze ICC was set to be carried out with an interval of two weeks since patients with chronic disease in this interval were considered to be in the same health state and conditions. Since patients with CAPD in our study visited the hospital on a monthly basis, test–retest for patients with CAPD (10 out of 40 participants) was one month apart from the baseline. To assure that patients were in the same health conditions between this 1 month interval, we confirmed that these patients did not experience any major changes in health state that could significantly affect the HRQOL, such as hospitalization. A statistical analysis showed no significant differences in all generic and kidney-specific domains between baseline and retest in the test–retest study.

Another limitation is that in the pilot testing, the interviews of patients to assure the clarity and interpretation were not recorded; therefore, content validity could not be performed quantitatively. The last limitation is that our study used a cross sectional design; therefore, we could not report responsiveness as part of the psychometric analysis. Further research is needed to examine the responsiveness to detect minimum changes in health status that are meaningful in clinical practice.

## Conclusion

The study supports the validity and reliability of both generic and kidney-specific domains of the KDQOL-36 Bahasa Indonesia to evaluate quality of life in patients with HD and CAPD treatments in Indonesia. Therefore, our study recommends the use of KDQOL-36 Bahasa Indonesia in dialysis centers to examine health-related quality of life, which can serve as a crucial predictor of patient outcomes.

## Supplementary Information

Below is the link to the electronic supplementary material.

Supplementary file1 (DOCX 2000 KB)
