# Supplementary material for: Psychometric properties of kidney disease quality of life-36 (KDQOL-36) in dialysis patients in Indonesia
Source: Qual Life Res. 2022 Aug 29;32(1):247–58. doi: 10.1007/s11136-022-03236-6 (PMC9829614; doi:10.1007/s11136-022-03236-6)
Supplement: Supplementary file 1 — Supplementary file1 (DOCX 2000 KB) [file 11136_2022_3236_MOESM1_ESM.docx]

# **Supplement 1.** Confirmatory Factor Analysis for the generic domains (SF-12) of KDQOL-36 Bahasa Indonesia

Confirmatory factor analysis (CFA) was evaluated by using the diagonally weighted least squares (DWLS) estimator in the lavaan package in R. CFA for the generic domains (SF-12) of KDQOL-36 Bahasa Indonesia was initially analyzed by including 2 factors/domains (PCS and MCS) without specifying the covariations between the error of the items that belong to the same subdomains. Each latent variable (domain) was allowed to correlate with one another. Variances for latent variables were set to 1, while loading factors on other domains were fixed to zero. In the lavaan package, by default, the scaling of the latent variable is achieved by fixing the loading of the first indicator (manifest variable) for a certain latent variable to the value of 1 (Figure S1). In addition, when the ordered= TRUE was used, lavaan would use diagonally weighted least squares (DWLS) to estimate the model parameters. In addition, by using ordered= TRUE, it was indicated that we analyzed ordinal/categorical data.

To visualize the results, we used the semPaths function in the semplot package (Figure S1). The standardized parameter estimates are displayed in Figure S2.


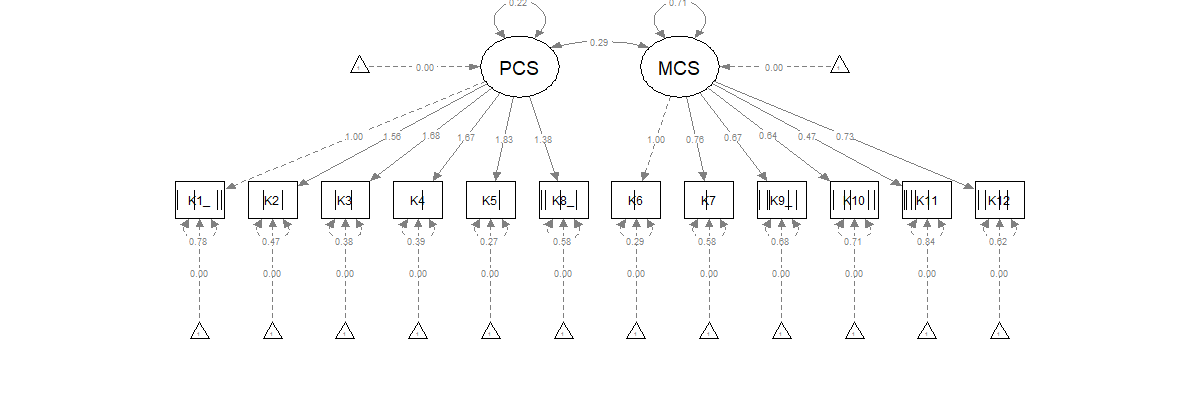


Figure S1. Visualization of the initial confirmatory factor analysis of the generic domains (SF-12) of KDQOL-36 Bahasa Indonesia using the unstandardized parameter estimates


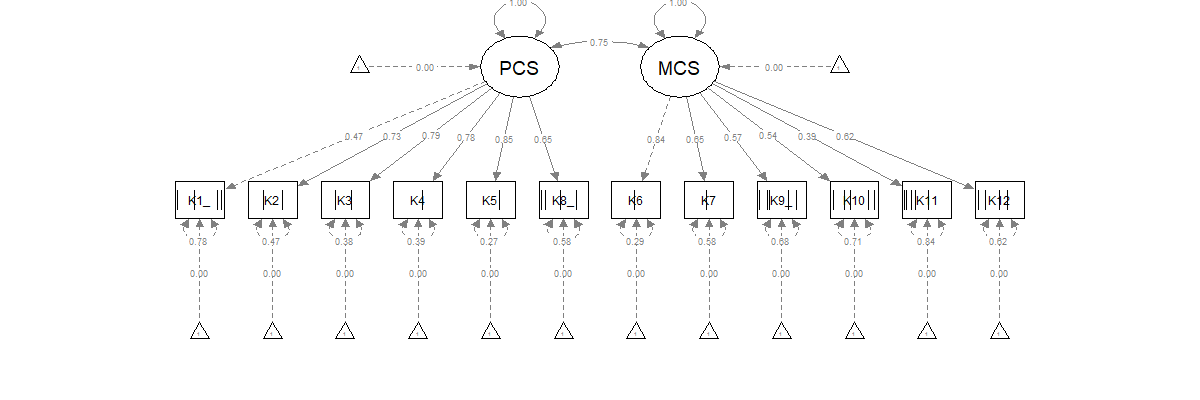


Figure S2. Visualization of the initial confirmatory factor analysis of generic domains (SF-12) of KDQOL-36 Bahasa Indonesia using the standardized parameter estimates

The goodness-of-fit for this model was χ2=204.92 (p-value <0.001), RMSEA value of 0.088, CFI of 0.958 and TLI 0.947.

After that, we re-analyze the CFA by adding the specific covariations between the error of the items that belong to the same subdomains. The parameters of goodness-of-fit were improved, which were indicated by an increase in CFI and TLI and a lower value of the RMSEA. In the manuscript, we displayed the standardized parameter estimators of the later model after the addition of specific covariations.

# **Supplement 2**. Confirmatory Factor Analysis for kidney-disease specific domains of KDQOL-36 Bahasa Indonesia

CFA was evaluated by using the diagonally weighted least squares (DWLS) estimator in the lavaan package in R. CFA for kidney-specific domains of KDQOL-36 Bahasa Indonesia was initially analyzed by including 3 factors/domains (burden, symptoms and effects of kidney disease). Each latent variable (domain) was allowed to correlate with one another. Variances for latent variables were set to 1, while loading factors on other domains were fixed to zero. In the lavaan package, by default, the scaling of the latent variable is achieved by fixing the loading of the first indicator (manifest variable) for a certain latent variable to the value of 1 (Figure S3). In addition, when the ordered= TRUE was used, lavaan would use diagonally weighted least squares (DWLS) to estimate the model parameters. In addition, by using ordered= TRUE, it was indicated that we analyzed ordinal/categorical data.

To visualize the results, we used the semPaths function in the semplot package (Figure S3). The standardized parameter estimates are displayed in Figure S4.


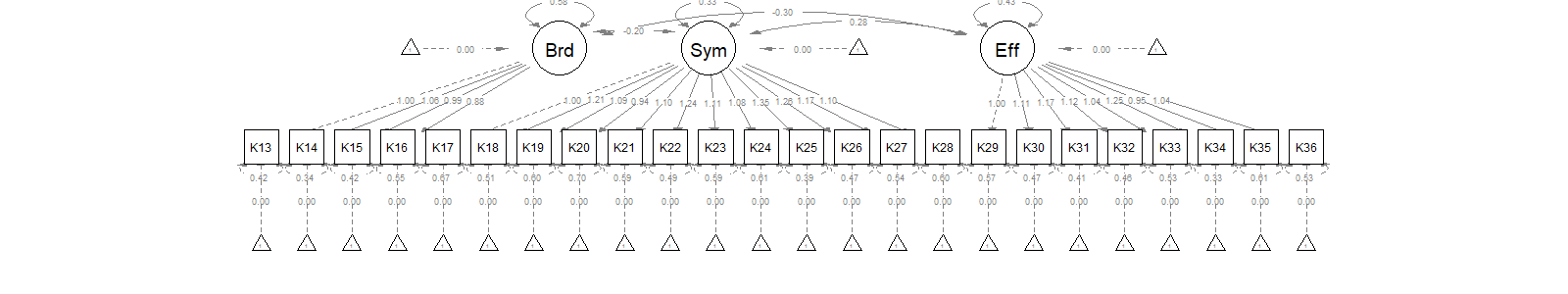


Figure S3. Visualization of the confirmatory factor analysis of the kidney-specific domains of KDQOL-36 Bahasa Indonesia using the unstandardized parameter estimates


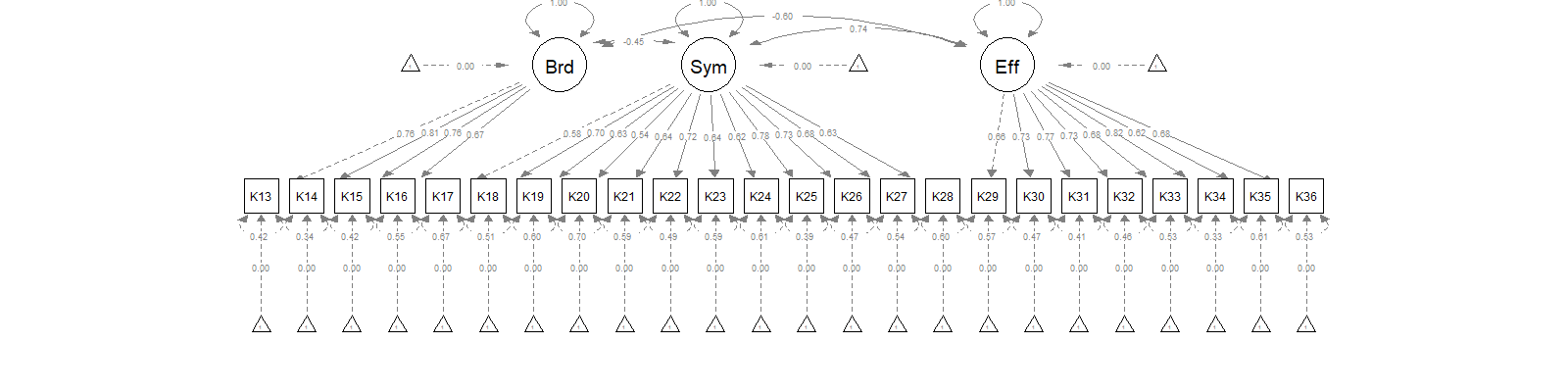


Figure S4. Visualization of the confirmatory factor analysis of kidney-specific domains of KDQOL-36 Bahasa Indonesia using the standardized parameter estimates

# **Supplement 3.** Exploratory Factor Analysis for kidney-disease specific domains of KDQOL-36 Bahasa Indonesia

Exploratory factor analysis (EFA) was conducted using the psych package in R, and the weighted least squared and polychoric correlations were used. The number of factors extracted for kidney-disease specific domains of KDQOL-36 Bahasa Indonesia was determined using the parallel analysis. The parallel analysis suggested 3 factors. The scree plot is displayed in Figure S5, while observed and random eigenvalues are presented in Table S1.


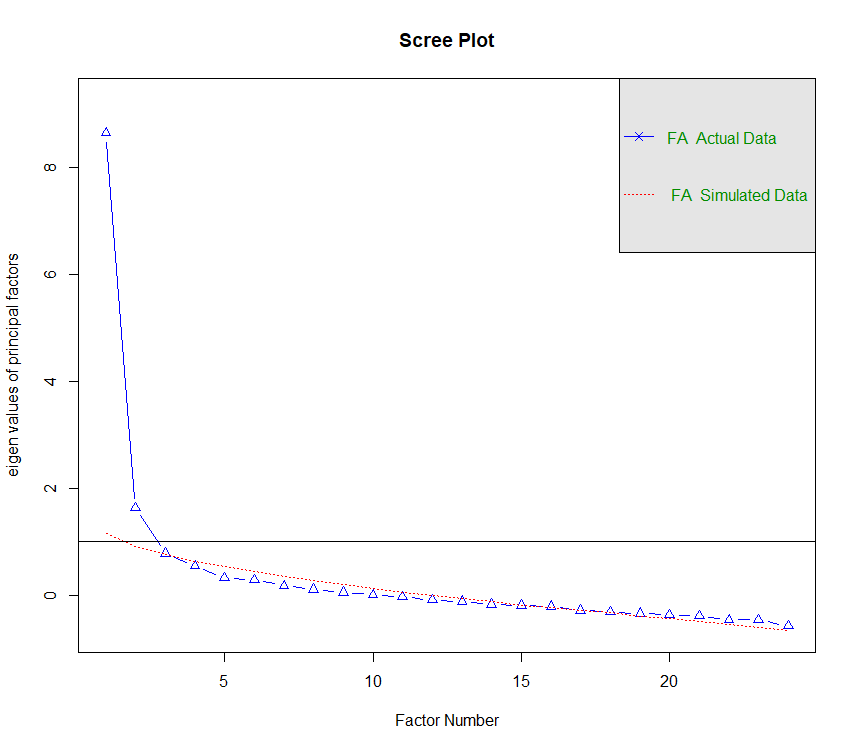


Figure S5. Parallel analysis to determine the number of factors to be extracted for kidney-disease specific domains of KDQOL-36 Bahasa Indonesia

Table S1. Observed and random eigenvalues from parallel analysis

| Factor | Observed eigenvalue | Random eigenvalue |
| --- | --- | --- |
| 1 | 8.64852318 | 1.158919189 |
| 2 | 1.64079321 | 0.859933865 |
| 3 | 0.78485088 | 0.744766087 |
| 4 | 0.54964713 | 0.624455881 |
| 5 | 0.33226728 | 0.519420653 |
| 6 | 0.29219340 | 0.435476438 |
| 7 | 0.17937078 | 0.354399516 |
| 8 | 0.11490583 | 0.286912813 |
| 9 | 0.05391353 | 0.204951449 |
| 10 | 0.01380049 | 0.139791521 |
